# Long non‐coding RNAs and their targets as potential biomarkers in breast cancer

**DOI:** 10.1049/syb2.12020

**Published:** 2021-05-15

**Authors:** Maryam Khalid, Rehan Zafar Paracha, Maryum Nisar, Sumaira Malik, Salma Tariq, Iqra Arshad, Amnah Siddiqa, Zamir Hussain, Jamil Ahmad, Amjad Ali

**Affiliations:** ^1^ Research Centre for Modeling and Simulation ‐ RCMS National University of Sciences and Technology (NUST) Islamabad Pakistan; ^2^ The Jackson Laboratory for Genomic Medicine Connecticut USA; ^3^ Department of Computer Science and Information Technology University of Malakand Chakdara Pakistan; ^4^ Atta‐ur‐Rahman School of Applied Biosciences ‐ ASAB National University of Sciences and Technology (NUST) Islamabad Pakistan

## Abstract

Breast cancer is among the lethal types of cancer with a high mortality rate, globally. Its high prevalence can be controlled through improved analysis and identification of disease‐specific biomarkers. Recently, long non‐coding RNAs (lncRNAs) have been reported as key contributors of carcinogenesis and regulate various cellular pathways through post‐transcriptional regulatory mechanisms. The specific aim of this study was to identify the novel interactions of aberrantly expressed genetic components in breast cancer by applying integrative analysis of publicly available expression profiles of both lncRNAs and mRNAs. Differential expression patterns were identified by comparing the breast cancer expression profiles of samples with controls. Significant co‐expression networks were identified through WGCNA analysis. WGCNA is a systems biology approach used to elucidate the pattern of correlation between genes across microarray samples. It is also used to identify the highly correlated modules. The results obtained from this study revealed significantly differentially expressed and co‐expressed lncRNAs and their *cis‐* and *trans‐*regulating mRNA targets which include RP11‐108F13.2 targeting TAF5L, RPL23AP2 targeting CYP4F3, CYP4F8 and AL022324.2 targeting LRP5L, AL022324.3, and Z99916.3, respectively. Moreover, pathway analysis revealed the involvement of identified mRNAs and lncRNAs in major cell signalling pathways, and target mRNAs expression is also validated through cohort data. Thus, the identified lncRNAs and their target mRNAs represent novel biomarkers that could serve as potential therapeutics for breast cancer and their roles could also be further validated through wet labs to employ them as potential therapeutic targets in future.

## 1 INTRODUCTION

Breast cancer (BC) is a genetically diverse disease and one of the major types of cancer. Based on statistical facts, approximately 1,675,000 women are affected by BC every year with a mortality rate of 500,000 [1]. One of the major reasons behind the prevalence of BC is its poor prognosis due to the high diversity demonstrated in its pathological features and behaviours [2]. Clinically, breast cancer is divided into four main molecular subtypes, Luminal‐A, Luminal‐B, HER2‐Positive and Triple negative breast cancer [3]. These subtypes are based on the hormonal and growth receptors that is oestrogen receptor (ER), progesterone receptor (PR) and human epidermal growth factor receptor2 (HER2) regulating cell growth signalling [2, 4]. Keeping in mind the pathogenic effects of this disease, there is a sheer need to identify novel therapeutic targets to combat BC [5]. Various studies provide evidence about the abnormal expression of lncRNA genes in breast tumours [6, 7]. They play an important role in regulating cancer pathways [8]. The size of lncRNAs is approximately greater than 200 nucleotides and they are the major regulators of numerous biological mechanisms, that is embryonic stem cell pluripotency, cell‐cycle regulation, different cellular pathways, gene expression regulation, transcription and post‐transcriptional regulation which have been found to be altered in various cancers. They regulate the transcription of protein coding genes by acting in a *cis‐* and *trans‐*manner [9, 10]. lncRNAs also exist as antisense transcripts in nature, affect the mRNA processing by influencing editing of pre‐mRNA and interrupt or inhibit mRNA splicing events, which results in affected or no proteins at all [11, 12]. They determine the total number of proteins produced by mRNA through regulation of post‐transcription activities. They also influence mRNA stability in both positive and negative manners [13, 14].

Various studies have reported the overexpression of lncRNAs in some breast cancer tissues [6, 9]. Based on their functions and expression patterns in tumour tissues, they can behave as oncogenes or tumour suppressor genes [15]. These studies have demonstrated the role of lncRNAs in mediating the aetiology of BC. [16]. This necessitates a systematic investigation to discover novel lncRNAs, along with their target mRNAs to aid the identification of novel biomarkers and potential therapeutic targets for BC [5]. Despite the major progress in lncRNAs discovery, they still lack in their annotation and functional characterisation [17, 18]. So it is essential to fill this gap through increased analysis and discovery of lncRNAs in various diseases. New workflows and methods in genomics research have paved the way for conducting systems wide analysis at different levels of diseases. Moreover, analysing lncRNAs which are both differentially expressed and co‐expressed will assist in discovering the genetic causes responsible for a particular disease [19] and co‐expression will provide significant biomarkers and therapeutic targets by integrating the significant gene expression correlation and their clinical outcome [7, 20]. Such an analysis will increase the functional importance of lncRNAs and will help in using them as diagnostic and therapeutic targets in future.

In this study, integrated statistical analysis was conducted on the expression profiles of lncRNA and mRNA data of breast cancer patients and normal samples. Differential expression (DE) analysis and co‐expression network analysis have been performed on these samples. A subset of both these datasets that can serve as significant biomarkers were obtained from the results. These biomarkers were then subjected to target identification to discover the novel lncRNA‐mRNA interaction that can further help in the retrieval of therapeutic targets. Their role in breast cancer was further confirmed through pathway analysis.

## 2 MATERIALS AND METHODS

**FIGURE 1:**
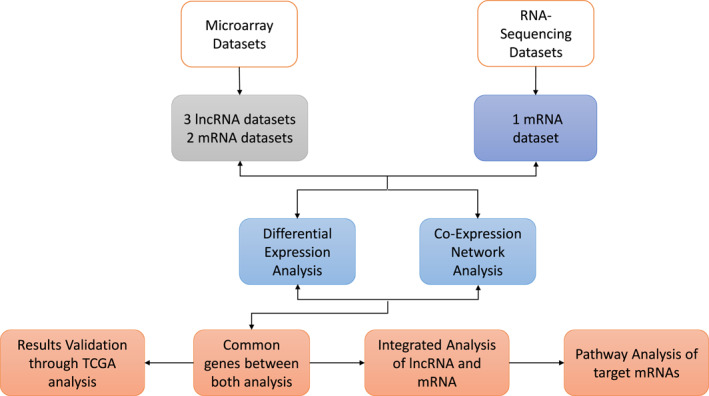
Overall Workflow of Methodology. Microarray and RNA‐Seq datasets were retrieved from the Gene Expression Omnibus (GEO) database. The total number of samples in microarray datasets were 10, whereas, RNA‐Seq data comprised 20 samples. Furthermore, microarray and RNA‐Seq data analysis was performed for the identification of differentially expressed lncRNAs. After that, co‐expression network analysis was performed on both datasets and common lncRNAs were selected from both types of analyses that is, DE analysis and co‐expression network analysis. Targets for the selected lncRNAs were found and merged with the mRNA subsets for retrieving common biomarker genes in breast cancer in integrated analysis

This study utilised BC samples to perform differential expression analysis of lncRNAs of microarray expression data and mRNA from RNA‐seq data. Co‐expression network analysis and pathway analysis of both samples were also performed. Moreover, integrated analysis was performed to identify the targets of lncRNAs in mRNA samples. The overall methodology of this study is shown in Figure 1.

### 2.1 Dataset selection

Dataset selection was based on experiments from human origin excluding the cell lines and samples which contain any mutations, drug and specific gene‐ or protein‐induced effects. Moreover, two datasets from both microarray and RNA‐Seq platforms were selected. These datasets contain an appropriate number of samples for both breast cancer patients and control group to generate a statistically significant analysis.

### 2.2 Microarray expression data

The quantile normalised series matrix file of the publicly available lncRNA microarray dataset GSE80266 was obtained from the GEO database. The genetic chip, which was processed by in situ oligonucleotide technology, comprised the GPL15314 Arraystar Human lncRNA microarray V2.0, (Agilent_033,010 Probe Name version). It contains a total of 10 samples including 5 breast cancer samples and 5 from the control group. The quality of data was assessed through the box plot and MA plot. Moreover, lncRNA analysis was performed on two other publicly available datasets of microarray platforms from the GEO database with accession numbers GSE113851 and GSE119233, respectively. The dataset GSE113851 comprised a chip with spotted oligonucleotide technology and the GPL16847 Invitrogen NCode Human Non‐coding RNA Microarray (NCRAH‐02) platform. Its sample size was 57 including 44 breast cancer and 13 normal breast samples. GSE119233 consists of in situ oligonucleotide technology with GPL16956 Agilent‐045,997 Arraystar human lncRNA microarray V3 (Probe Name Version). It contains 30 samples including 10 normal and 20 breast cancer samples.

### 2.3 Re‐annotation and differential expression analysis of lncRNAs

The series matrix file of dataset GSE80266 represented the expression levels of probes with the probe IDs. To find the respective genes, re‐annotation was performed for the retrieval of the corresponding Ensemble IDs. For this purpose, the probe sequences were mapped to human reference genome hg38 using TOPHat [21], and converted to an identifiable browser extensible format (BED) utilising BED tools [22]. The probes with a mapping score of 50 were considered significant. A coding potential assessment tool (CPAT) [23] was used to filter the coding and non‐coding sequences.The non‐coding sequences were subjected to the Biomart package of R software for accessing their Ensemble IDs and gene names.

The difference in expression levels was measured by comparing the control with the case samples, applying the Student’s *t*‐test along with a multiple test correction method by Benjamini‐Hochberg [24]. The decision rule for selecting differentially expressed lncRNA genes was based on both p‐value and adjusted p‐value less than 0.05. The filtered non‐coding probes and lncRNA DEGs are given in the supplementary file 1.

For both datasets used for validation, DEG analysis was performed on series matrix files. The quality assessment was performed using box plots and log2 transformation [25] was applied for normalisation. DE analysis was performed using limma [26] and re‐annotation of the DEGs for dataset GSE119233 was retrieved from BLAT [27], while annotation for GSE113851 was already provided with the dataset.

### 2.4 RNA‐seq analysis

Transcriptome studies on eukaryotes have been upgraded with the advancements in next‐generation sequencing technologies [28]. RNA‐sequencing is one of the important NGS techniques used in transcriptome analysis, which have exceeded the microarray technologies, as they use highly parallel methods that are less time consuming and more predictive [29]. It has great importance in the identification of gene expression profiles, novel gene discovery, finding alternative variants, transcripts and allele specific expressions, etc. and has provided an unlimited knowledge based on these studies [30].

The publicly available RNA‐Seq dataset GSE52194 processed form Illumina HiSeq was obtained from GEO, containing 17 breast cancer samples with three different subtypes (6 = triple negative, 6 = non‐triple negative, 5 = HER2 positive) and 3 control samples. The paired end raw data files were retrieved in FATSQ format. The quality of data was assessed using FASTQC tool [31] and technical errors were preprocessed using Trimmomatic [32]. For preprocessing, ILLUMINACLIP, SLIDINGWINDOW, LEADING and TRAILING were used with their default settings. HEADCROP, which removes a specific number of bases from the start of read, was given the value 15 for the removal of contaminated bases in the data, and MINLEN which removes the read if it falls off a threshold length was given the value of 36 [32]. After preprocessing, errors were removed from the data. The preprocessed sequences were mapped to the human reference genome hg38 with Bowtie [33]. These mapped sequences were analysed using featureCounts tool. This whole analysis was performed employing a pipeline of tools built on the galaxy platform [34].

### 2.5 Differential expression analysis of mRNA data

Three different R tools, that is DESeq [19], edgeR [35] and limma‐voom [26] were utilised for determining the differential expression of mRNA read counts to acquire the best results with significant p‐value and the false discovery rate (FDR) of threshold <0.05. For DE analysis, the read counts with low expression values were removed. Genes with the minimum 1 counts per million (cpm) in at least two samples were kept for further analysis. The read counts were normalised through trimmed mean of M values (TMM) normalisation, applied by the edgeR and limma‐voom in R, while DESeq, applied median of ratios for normalisation of the counts. Filtered expression matrix for DE analysis of mRNA is provided in supplementary file 2.

For assuring the significance of analysis and differential expressions of target mRNA genes in BC, another DEGs analysis was performed on two publicly available mRNA datasets from GEO database with microarray platforms having accession numbers GSE22820 and GSE26910, respectively.

The genetic chip of GSE22820 was processed by in situ oligonucleotide technology and comprised GPL6480 Agilent‐014,850 Whole Human Genome Microarray 4 × 44 K G4112 F (Probe Name version). Its sample size was 186 including 10 samples from control group and 176 from primary breast cancer patients. Moreover, the dataset GSE26910 consists of in situ oligonucleotide chip with GPL570 [HG‐U133‐Plus‐2] Affymetrix Human Genome U133 Plus 2.0 Array. It consists of a total of 24 samples, including 6 control and 6 breast cancer samples along with 6 samples from control group and 6 prostate cancer samples; from this whole dataset, only 12 samples with BC patients along with their respective controls were used for performing DE analysis.

Differential expression analysis of both datasets was performed on their series matrix files. The quality of data was observed using box plots. For normalisation, log2 transformation [25] was performed on both datasets and significantly differentially expressed genes were retrieved using limma [26] from R Bioconductor package.

### 2.6 Analysis of Co‐expression networks

The co‐expression network analysis facilitates in finding the clusters of highly correlated or co‐expressed gene expression profiles and helps to determine the candidate biomarkers and potential therapeutic targets of various diseases including cancer. This analysis was performed using WGCNA package [20] in R, to acquire the novel biomarkers that could act as breast cancer therapeutics as it provides the cluster of hub genes involved in similar disease pathways [20]. Quantile normalised microarray data and RNA‐Seq data with vst and quantile normalisation was utilised for analysis. An initial unsupervised hierarchical clustering revealed no outliers in both types of data. The soft threshold used for microarray data was 6 and that for RNA‐Seq data was 9. An unsigned network was built on the basis of Pearson correlation with p‐value <0.05 for significantly correlated modules. The threshold for minimum module size was 30 and module membership was built for analysis of significant genes highly correlated with breast cancer.

### 2.7 Identifying lncRNA‐mRNA interactions

For the retrieval of lncRNA‐mRNA interactions, common lncRNAs were selected from both differential expression analysis and WGCNA analysis. For verifying the chromosomal locations of selected genes, an alignment tool Blat was used [27]. Those positions were further used to retrieve the gene IDs and names from BioMart along with their upstream and downstream target genes. Moreover, R‐code was developed, considering the *cis‐* and *trans‐*activity of lncRNA, for retrieving the target genes from selected lncRNAs. It was based on the assumption that the *cis‐* and *trans‐*lncRNA target genes are located within the locality of 10‐300 kb away from lncRNAs in the upstream or downstream region, respectively [36]. After the retrieval of lncRNA target genes, they were merged with mRNA genes in R. A subset of common genes retrieved after merging were determined as differentially expressed biomarker mRNA genes. Their role in breast cancer was also confirmed through pathway analysis using different databases including KEGG [37], PathCards [38] and literature search.

### 2.8 TCGA of mRNA

To verify the involvement of mRNA targets of lncRNAs in breast cancer, TCGA cohort was performed using Bioconductor R package (TCGAbiolinks) [39]. The total 140 breast cancer primary tumour samples and 113 normal breast tissues samples were retrieved from TCGA database. The quantile normalisation was performed on data and then mRNA differential expression analysis was preformed utilising Bioconductor egdeR [35]. Then *Log*_2_ *FC* values of target mRNAs were analysed.

The http://gepia.cancer‐pku.cn/GEPIA (http://gepia.cancer‐pku.cn/) tool was also used to validate the expression of mRNA targets in breast cancer. A total of 1085 tumour samples and 291 normal were analysed. LIMMA method for differential expression analysis was employed to determine the expression values.

## 3 RESULTS

### 3.1 Quality analysis of microarray data

Box plot and MA plots were plotted to analyse the quality of quantile normalised microarray data. The overall quality of data was good and plots revealed that data does not contain any outliers.

### 3.2 Identification of differentially expressed lncRNAs

The total number of probes was 34,576 and they were reduced to 30,627, after filtering on the basis of mapping score. These probes were then passed through a tool, CPAT, which gave 29,797 non‐coding RNAs and 830 mRNAs. These 29,797, non‐coding probes were employed for further analysis. By applying *t*‐test, 181 significantly differentially expressed lncRNA genes were obtainedand selected on the basis of p‐value and adjusted p‐value <0.05. Supplementary file 3 provides the lncrna DEGs.

A total 14,482 DEGs were retrieved from lncRNA dataset GSE113851 and 8400 DEGs were provided by GSE119233. After detailed analysis of the results from both of the lncRNA datasets, it was observed that they did not provide the exact locations of the lncRNAs discovered in this study. The major reason is that the dataset GSE80266 used in analysiscomprised novel long non‐coding RNAs in breast cancer and the data was without any specific conditions and its re‐annotation was carried out on the criteria of selecting the sequence with the highest scoring. Moreover, annotations for one of the datasets used for validation GSE113851 were provided already and the other dataset GSE119233 was from Basal‐like Breast Cancer, while we were focusing on the generalised BC dataset.

### 3.3 Examining differentially expressed mRNA

**FIGURE 2:**
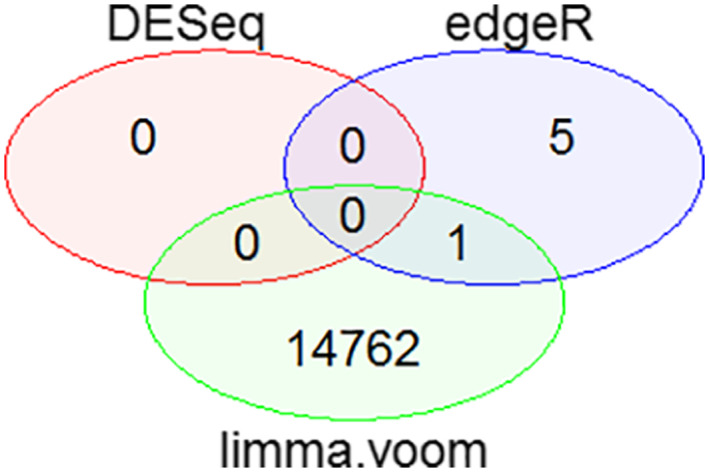
Venn diagram for the Comparison of DESeq, edgeR and limma‐voom on the Basis of p‐value. It shows the total number of significant genes observed from each tool based on p‐value < 0.05. No significant differentially expressed genes (DEGs) were obtained from DESeq. EdgeR. It gave five significant DEGs based on p‐value and limma‐voom provided 14,762 significant differentially expressed genes on the basis of both p‐value and adjusted p‐value. There was 1 common DEG between edgeR and limma‐voom

The preprocessed and normalised read counts were analysed for differential expression analysis using three different tools in R that is, DESeq, edgeR and limma‐voom. DESeq when tested on first subtype that is Triple negative breast cancer did not give the significant p‐value and adjusted p‐value, that is <0.05 for a single gene count due to which its results were not considered. Moreover, edgeR gave few DEGs with significant p‐value. But when the false discovery rate (FDR) correction was applied on edgeR, it did not provide any significant results on the basis of p‐adjusted value. After that, limma‐voom was applied and it outperformed in providing significant results for a large number of gene counts on the basis of both p‐value and adjusted p‐value less than 0.05. So it was used further for all the subtypes of breast cancer. The overall comparison of these tools on the basis of p‐value is shown in Figure 2.

These results were in accordance with a study which reported that limma‐voom provides better results under different conditions, which remains least affected by outliers, works faster computationally and requires at least three samples to perform the analysis [40]. The total number of significant DEGs obtained for TNBC subtype was 7113 with upregulation, 2 DEGs were from non‐TNBC with down regulation and 16,988 DEGs were from HER2 positive subtype including 16,882 upregulated and 106 downregulated genes. Significant DEGs of the three subtypes of BC is provided in [Link], [Link] and 6, respectively.

Moreover, the datasets used for validation of analysis provided a total of 17,423 DEGs which were obtained from GSE22820 and 25,000 DEGs were retrieved from GSE26910.

### 3.4 Co‐expression analysis using WGCNA

**FIGURE 3:**
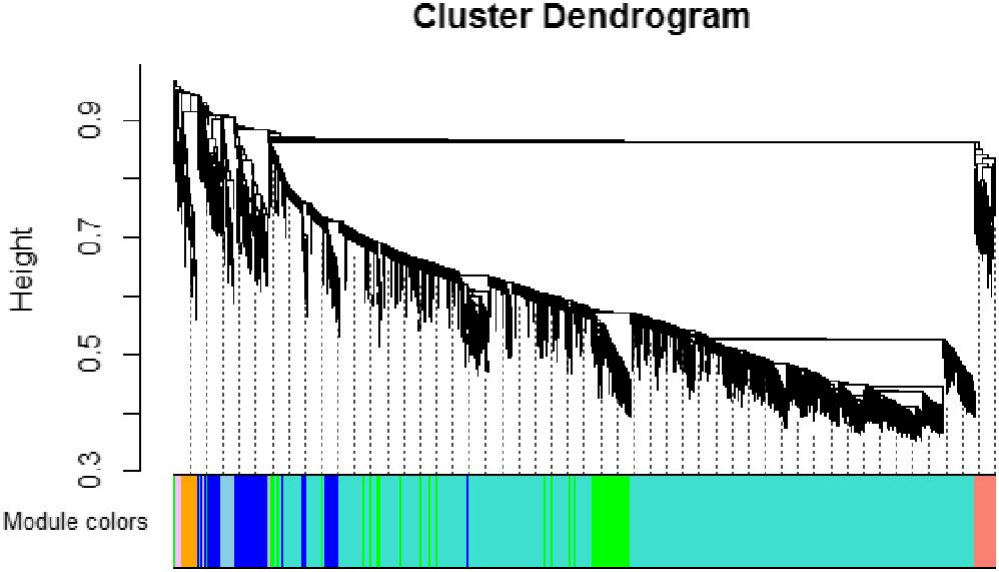
Cluster Dendrogram of lncRNA modules dataset GSE80266. The branches of the clusters represent modules with correlated genes, distinguished from each other on the basis of different colours

The co‐expression network analysis when executed on microarray data of which built a cluster dendrogram based on unsupervised hierarchical clustering method, illustrated in Figure 3.

**FIGURE 4:**
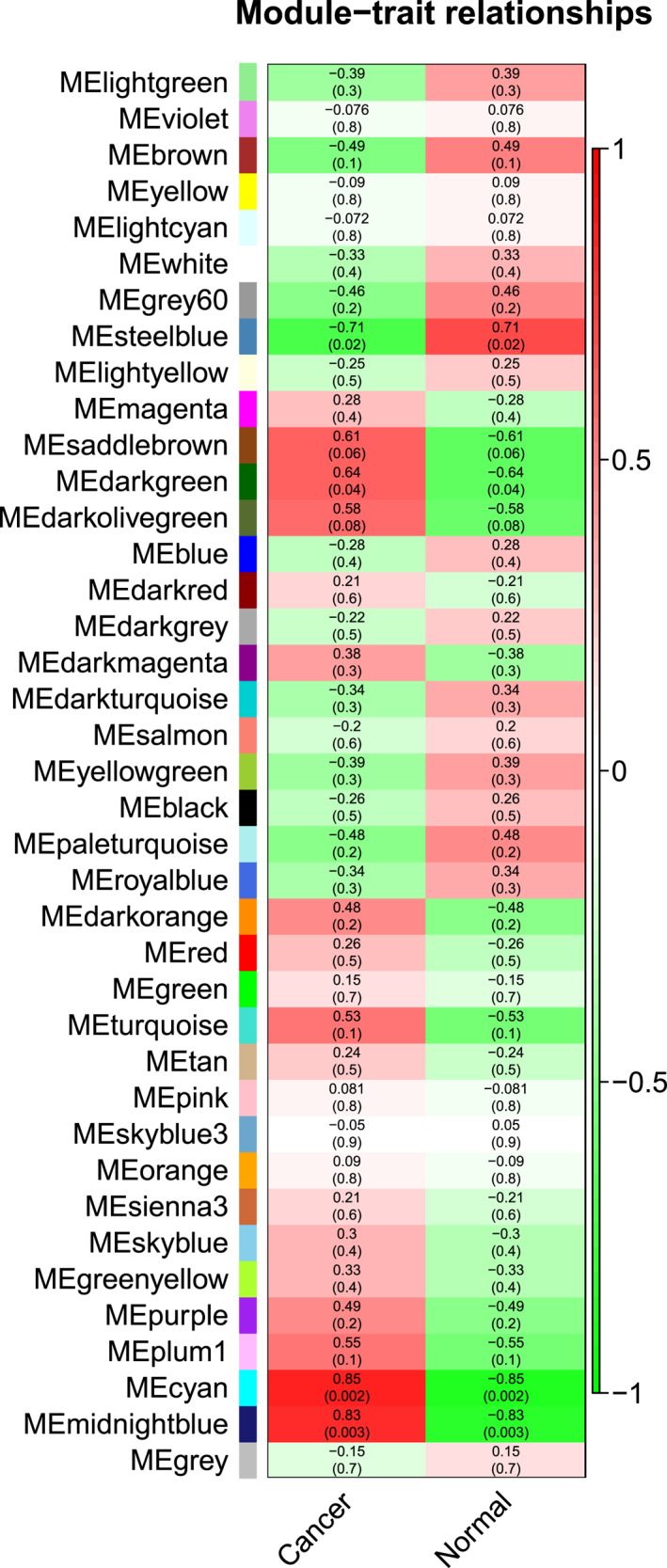
Module‐Trait relationship of lncRNAs dataset GSE80266. There are 39 modules, each represented with a colour name along with their correlation and p‐values. Modules with dark red colour in the heat map, that is MEcyan and MEmidnight blue show a high correlation of 85% and 83% along with their significant p‐values 0.002 and 0.003, respectively

A module‐trait relationship built with traits, that is breast cancer and normal, provided 39 modules in total on the basis of significant correlation approaching to ±1 and a threshold p‐value <0.05. The modules are shown with their correlation and p‐values in the Figure 4.

Two modules MEcyan and MEmidnight blue showed a significant correlation of 85% and 83% along with p‐values 0.002 and 0.003, respectively. Furthermore, MEcyan module comprised 376 total genes out of which 127 were significantly correlated with breast cancer and MEmidnight blue included 193 lncRNA genes with 140 genes having a significant correlation with cancer. Therefore, a total of 267 lncRNAs obtained from WGCNA were significantly correlated with cancer. Particulars for all modules from WGCNA analysis of lncRNA are given in supplementary file 7.

Moreover, WGCNA analysis was also performed on two more lncRNA datasets GSE113851 and GSE119233 from the microarray platform. The cluster dendograms for both the datasets are illustrated in supplementary Figures 6 and 8.

Moreover, two traits that is breast cancer and normal were used to built a module‐trait relationship for both datasets. For dataset GSE113851, the module‐trait relationship provided a total of 75 modules based on significant correlation approaching to ±1 and a threshold p‐value <0.05. The modules along with their correlation and p‐values are shown graphically in the supplementary Figure 7.

The module MEblue showed a high correlation with a significant p‐value and includes 3529 genes significantly correlated with breast cancer. The details of its WGCNA analysis are given in supplementary file 8.

For dataset GSE119233, a total of 14 modules were retrieved through module‐trait relationship based on significant correlation approaching ±1 and a threshold p‐value <0.05. The modules along with their correlation and p‐values are shown graphically in the supplementary Figure 9.

The module MEblue includes 235 genes significantly correlated with breast cancer.The particulars of its WGCNA analysis are given in supplementary file 9.

**FIGURE 5:**
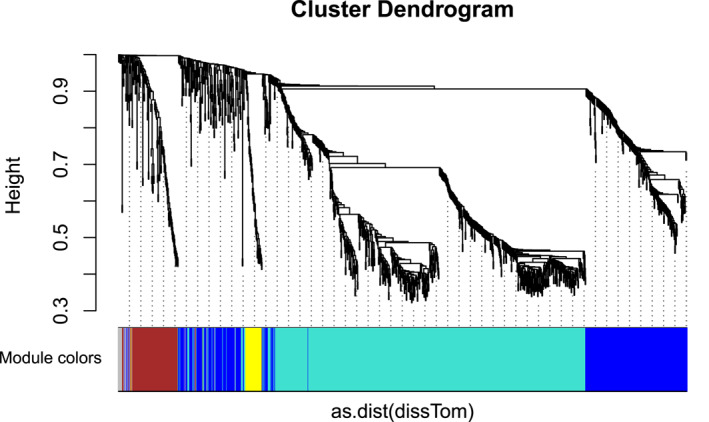
Cluster Dendrogram of mRNA modules dataset GSE52194. Branches of the dendrogram correspond to modules with correlated genes represented by different colours

**FIGURE 6:**
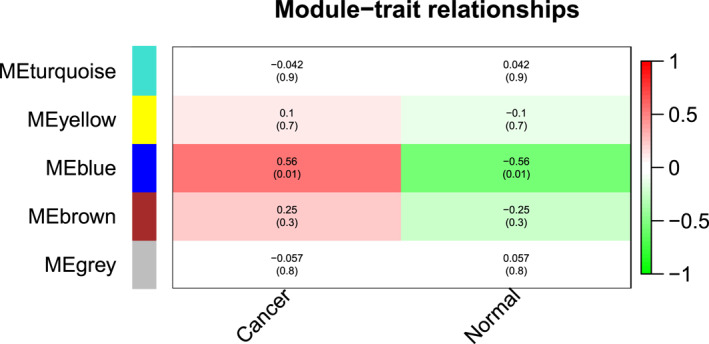
Module‐Trait relationship of mRNA modules dataset GSE52194. The heat map shows a total of five modules, generated by applying the correlation with external traits, that is cancer and normal. Moreover, MEblue was the only module shown in dark red colour to hold a 56% correlation with a least p‐value of 0.01

For mRNA data from RNA‐Seq analysis, the cluster dendrogram built is represented graphically in Figure 5. The module‐trait relationship gave 5 modules and only one module showed a significant correlation with a p‐value <0.05. The heat map built for visualising this relationship is shown in Figure 6. The MEblue provided maximum correlation of 56% with a least p‐value of 0.01. There were 339 genes in this module out of which 138 showed significant correlation with breast cancer (Supplementary File 10).

WGCNA analysis was also performed on two mRNA datasets GSE22820 and GSE26910 from the microarray platform for observing the co‐expression networks. For mRNA datasets GSE22820 and GSE26910, the cluster dendograms built are represented in supplementary Figures 10 and 12.

The module‐trait relationship for GSE22820 gave 15 modules and only one module that is MEmagenta showed a significant correlation of 58% with a least p‐value 2e −18. There were 71 genes in this module significantly correlated with breast cancer. The modules along with their p‐values and correlation are depicted in a heat map in supplementary Figure 11.

Moreover, the heat map of the module trait relationship of GSE26910 is illustrated in the supplementary Figure 13. The module MEmagenta and MEred showed a highest correlation of 90% each with significant p‐values of 6e‐05 and 7e‐05, respectively. MEmagenta includes 415 genes and MEred comprises 632 genes significantly correlated with BC. The particulars of WGCNA analysis for both datasets that is GSE22820 and GSE26910 are given in supplementary files 11 and 12, respectively.

### 3.5 Integrative analysis

**FIGURE 7:**
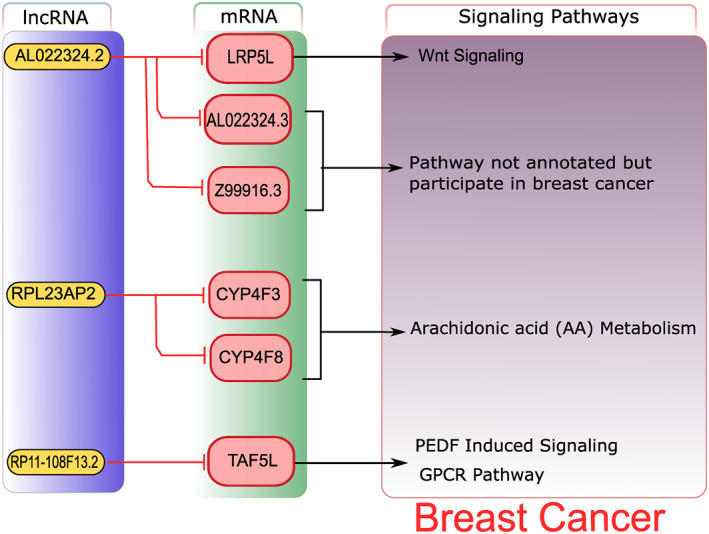
Overall Representation of Integrative analysis. Yellow coloured boxes represent lncRNAs and pink coloured boxes represent their *cis‐* and *trans‐*regulating mRNA targets. Red coloured lines with heads represent targets whereas arrows represent the constituent of the relevant signalling pathways. The lncRNA RP11‐108F13.2, based on analysis, targets mRNA gene TAF5L, which regulates basal transcription factors pigment epithelium‐derived factor induced signalling and the GPCR) pathway, RPL23AP2 was found to target CYP4F3, CYP4F8 genes which regulate and Arachidonic acid(AA) metabolism and lncRNA AL022324.2 was observed to target LRP5L, AL022324.3 and Z99916.3, respectively Among them LRP5L acts in the regulation of the Wnt signalling pathway and the breast cancer pathway. While AL022324.3 and Z99916.3 were found as breast cancer targets, their complete annotation was not found. GPCR, G‐protein coupled receptorPathway analysis through different databases such as PathCards [38], KEGG [37] and literature search of the genes retrieved through integrative analysis was performed. A brief illustration of the pathways regulated by target genes is shown in Figure 8

The integrative analysis was carried out after retrieving lncRNA and mRNA genes from WGCNA and DEGs analysis. A subset of three common lncRNAs including AL022324.2, RPL23AP2 and RP11‐108F13.2 was obtained from the whole analysis, their *cis‐* and *trans‐*regulating targets were acquired using the R code from Biomart package. The lncRNA targets retrieved, were merged with the subsets of common mRNA genes which were also significantly differentially expressed and co‐expressed. A total of six common targets were identified in mRNA subsets, including TATA‐Box Binding Protein Associated Factor 5 Like (TAF5L), Cytochrome P450 Family 4 Subfamily F Member 3 (CYP4F3), LDL Receptor Related Protein 5 Like (LRP5L), AL022324.3, Z99916.3 and Cytochrome P450 Family 4 Subfamily F Member 8 (CYP4F8). Upon further data analysis, it was determined that TAF5L, CYP4F3, LRP5L and AL022324.3 belong to both TNBC and HER2 positive subtypes while, Z99916.3 and CYP4F8 were just from HER2 positive subtype. Moreover, there were no common target genes from non‐TNBC subtype of BC in this particular dataset. Pathway analysis performed for ascertaining the roles of these target genes in breast cancer indicated that they regulate classic biological pathways which are crucial in triggering breast cancer. They regulate metabolic and signalling pathways, respectively. A brief illustration of this analysis is provided in the Figure 7.

**FIGURE 8:**
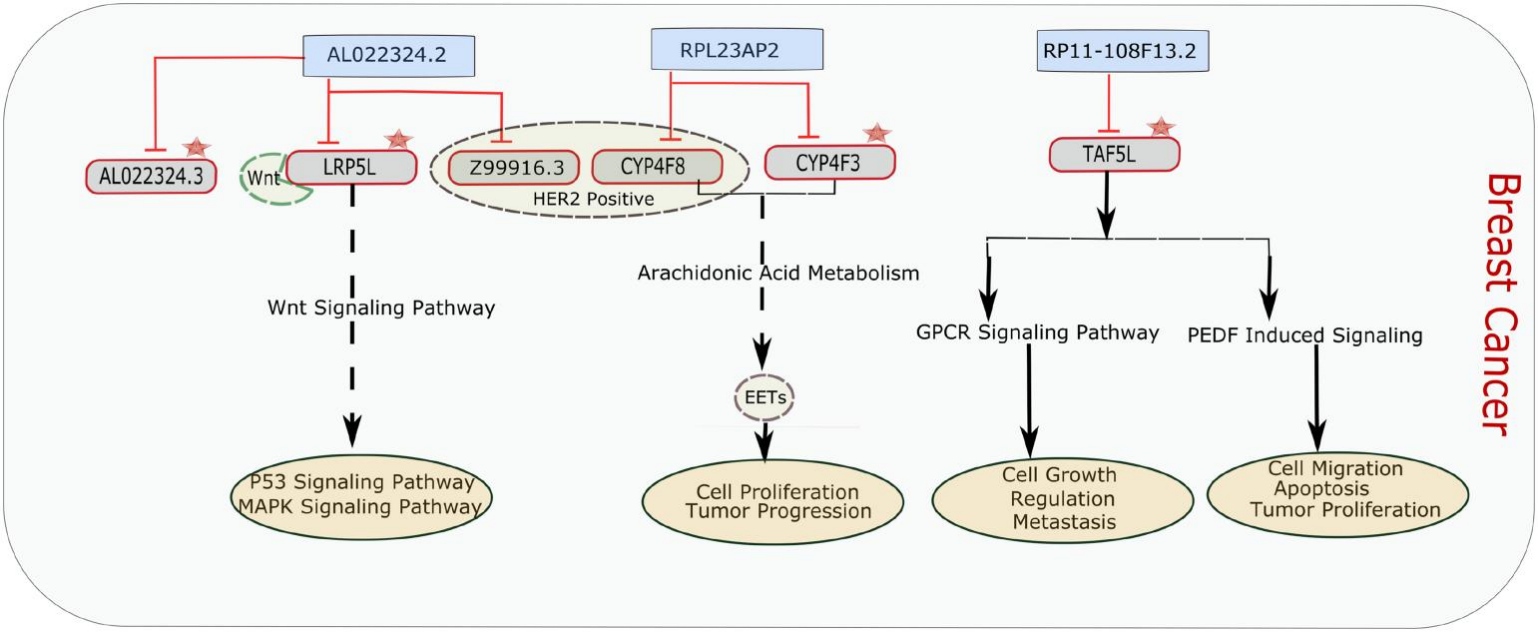
Brief Description of Targets and their Pathways. Blue coloured boxes represent lncRNAs and grey coloured boxes represent their *cis‐* and *trans‐*regulating mRNA targets. Red coloured lines with heads represent targets and arrows represent their relevant signalling pathways. The oval pink coloured boxes represent cellular mechanisms, regulated by these pathways. The dotted circle represents the target genes which are present in the HER2 positive subtype whereas the red star represents target genes common in both TNBC and HER2 positive subtype. LRP5L regulates the Wnt signalling pathway by binding to the Wnt ligand. This pathway has a crucial role in breast cancer. AL022324.3 and Z99916.3 were found to be breast cancer targets, but their pathways are not annotated yet. CYP4F3 and CYP4F8 genes regulate arachidonic acid (AA) metabolism and transform arachidonic acids to epoxyeicosatrienoic acid (EETs) which are involved in angiogenesis and contribute to the tumour progression. TAF5L regulates pigment epithelium‐derived factor (PEDF) induced signalling and the G‐protein coupled receptor (GPCR) pathway which are also the major contributors to the breast cancer pathways

According to studies, TAF5L which regulates basal transcription, and helps in transcription initiation by regulating transcription factors activity, is found to participate in PEDF‐induced signalling and the GPCR pathway [38]. PEDF in the normal state plays a potential role in tumour inhibition by its anti‐angiogenic function [41]. The down regulation of PEDF was observed overall in cancer, as its anti‐tumour activity may diminish during tumour proliferation. Different studies have also reported lower levels of PEDF in breast tumours, as it plays an inhibiting role in tumour angiogenesis and controls cellular differentiation [41, 42]. The GPCR pathway is comprised of G‐protein coupled receptors which are transmembrane receptors, controlling signal transduction pathways [43]. Cancer studies have found that GPCR plays a significant role in oestrogen‐negative (ER negative) breast cancers [44]. The mechanism of GPCR involved in this cancer is the indirect activation of epidermal growth factor receptor (EGFR), by the release of epidermal growth factor (EGF) ligands from the surface which activates the intracellular signalling pathways [44]. Such an activated mechanism may result in increased proliferation and tumour cell progression [45].

LRP5L was analysed through different studies to participate in the Wnt signalling pathway. It functions in Wnt‐protein binding and Wnt‐activated receptor activity. It has a major role in carcinogenesis and tumour progression [46, 47].

The target genes CYP4F3 and CYP4F8 were found to regulate fatty acids/lipid metabolism [48]. The metabolic reprogramming resulted due to gene mutations and epigenetic changes during cellular transformation, plays a key role in tumour cell development and progression. Proliferation and metastasis are preserved in cancer cells by enhancing the aerobic glycolysis and fatty acid synthesis [49, 50, 51].

The list of mRNAs identified in our study was also validated by TCGA and GEPIA breast cancer cohort data. The mRNA targets TAF5L and LRP5L were identified from TCGA data, their mean expression in tumour and normal cells, *log*_2_ *FC* and p‐value is provided in Table 1. Also, the breast cancer cohort data from GEPIA web‐based database and tool was analysed to identify target mRNAs. CYP4R3, TAF5L and LRP5L were retrieved (Table 1).

**TABLE 1:** TCGA and GEPIA results: The list of Target mRNAs identified from TCGA and GEPIA data

Sr. No	mRNAs	Mean Expression in tumour	Mean Expression in Normal	*log*_2_ *FC*	*p*‐value
TCGA results					
1	TAF5L	2540.679	1697.212	0.43842	5.70E‐14
2	LRP5L	238.1071	200.7965	0.177151	0.115538
GEPIA results					
1	CYP4F3	0.100	0.090	0.013	7.16e‐6
2	TAF5L	16.381	12.300	0.386	7.34e‐25
3	LRP5L	2.740	6.520	−1.008	3.83e‐70

Abbreviations: CYP4F3, Cytochrome P450 Family 4 Subfamily F Member 3; LRP5L, LDL Receptor Related Protein 5 Like; TAF5L, TATA‐Box Binding Protein Associated Factor 5 Like.

Thus, pathway analysis suggests that the signalling and metabolic pathways identified play a vital role in breast cancer regulation. Further investigation of these pathways could help in breast cancer therapy.

The DEGs obtained from the analysis performed for validation were further analysed for retrieving target mRNA genes obtained from our study and it was observed that GSE22820 includes TAF5L and CYP4F3 while GSE26910 involves AL022324.4, a contig sequence from one of the target genes AL022324.3 among significantly differentially expressed genes of breast cancer.

The WGCNA analysis was also performed for both mRNA and lncRNA datasets to analyse the presence of biomarker genes in our research. The subsets of common genes from DEG analysis and WGCNA were observed among all datasets;but specifically, the biomarker genes from our study were not discovered from the results of WGCNA analysis.

## 4 DISCUSSION

Both coding and non‐coding RNAs have been demonstrated as the key regulators with various biological functions. 90% of the human genome is transcribed into non‐coding RNAs and also play an important role in the development and progression of various types of cancers [52]. Breast cancer is one of the lethal types of cancer and is largely regulated by lncRNAs and mRNA [53, 54]. The aim of this study was to determine candidate biomarker genes and potential therapeutic targets that could aid in breast cancer therapy in the future. This study was conducted on breast cancer data of lncRNA and mRNA from two different platforms, that is microarray and RNA‐Seq. The objective of the study was to observe the differential expression patterns, co‐expression networks and pathway analysis of both differentially expressed and co‐expressed genes identified in breast cancer. The methodology used for this analysis includes microarray analysis, RNA‐Seq analysis, DE analysis, co‐expression network analysis (WGCNA) and integrative analysis of lncRNAs along with their *cis‐* and *trans‐*regulating target genes, which were integrated with identified mRNA genes and their pathway analysis. Three common lncRNAs were identified to be significantly differentially expressed and co‐expressed with the breast cancer genes, which include RP11‐108F13.2, RPL23AP2 and AL022324.2. The RP11‐108F13.2 which was reported to be an lncRNA [55] targets the mRNA gene, TAF5L. Through further analysis processed pseudogene of ribosomal protein L23a (RPL23AP2) was identified and its targets were CYP4F3, CYP4F8. Another processed pseudogene AL022324.2 obtained from results targets LRP5L, AL022324.3 and Z99916.3, respectively. The targets such as TAF5L, CYP4F3, LRP5L and AL022324.3 were common in both TNBC and HER2 positive subtypes, while the remaining two that is Z99916.3 and CYP4F8 were identified in HER2 positive subtype only and no significant DE lncRNA or mRNA targets were observed in non‐TNBC subtype.

The functional or molecular annotation of lncRNAs identified in this study was very limited or not reported and was determined only in some genomic databases being reported only as lncRNAs or pseudogenes with incomplete annotation. There is still a gap in the molecular and functional annotation of the lncRNAs which is required to be filled for better and improved understanding of functional properties and regulatory roles of lncRNAs in normal and diseased states, particularly in cancers. Aberrantly expressed mRNA genes were further examined through functional annotation and pathway analysis. They were identified to be involved in different biological processes such as metabolic and signalling pathways. All these pathways were determined as the major contributors in cancer progression and metastasis.

TAF5L gene was discovered through GO annotations to regulate basal transcription, and helps in transcription initiation by regulating promoter recognition and transcription factor activity and also acts in histone acetyltransferase activity [56]. It encodes for a protein which is the member of WD‐repeat of the TAF5 family of proteins. The encoded protein belongs to the PCAF histone acetylase complex [56] and comprises 20 different types of (TAF) polypeptide units and regulates cell cycle progression, transcription, and cellular differentiation [56]. A study has reported that PCAF is responsible for the dysregulation of phosphatase and tensin homologue deleted on chromosome 10 (PTEN) protein, which results in the tumour progression in breast, lung, prostate and brain as PTEN is the tumour suppressor gene [57]. According to a study, TAF5L was reported as a biomarker in breast cancer [58]. It regulates the PEDF‐induced signalling and GPCR pathways [38]. PEDF‐induced signalling plays a potential role in tumour inhibition and regulates anti‐angiogenesis [41]. The GPCR pathway regulates signal transduction and activates EGFR indirectly in cancer resulting in tumour progression and proliferation. Its role was also reported in ER‐negative breast cancer [43, 44]. The overexpression of TAF5L was reported in a study [58], which further affirms the upregulation of TAF5L as determined in our study. The upregulation TAF5L in breast cancer is also validated from the results of TCGA and GEPIA analysis with the positive Log2FC values which are provided in supplementary file 12. These results support the identification of TAF5L in this study and suggest that this gene could be used as a potential biomarker in breast cancer diagnosis and therapy.

The genes CYP4F3 and CYP4F8 encode for proteins which belong to the super family of cytochrome P450 enzymes [48], regulating several important biological processes, including cholesterol, lipids and steroids synthesis and metabolism [48]. Over expression of CYP4F3 and CYP4F8 was reported in several studies to regulate tumour progression and proliferation in various types of cancers such as breast and prostate cancer [59, 60]. They also regulate arachidonic acid (AA) metabolism [48]. It was determined that the metabolic reprogramming resulted due to gene mutations and epigenetic changes during cellular transformation plays a key role in tumour cell development and progression [61]. Cancerous cells maintain their proliferation and other metastasis activities by enhancing the aerobic glycolysis and fatty acid synthesis [49, 50, 51]. Based on these findings, the upregulation of CYP4F3 and CYP4F8 in breast cancer can be confirmed along with their role in tumour progression as their overexpression was also identified in our study.

The role of LRP5L was reported to regulate Wnt protein binding and to activate its receptors. It regulates the Wnt signalling pathway [62]. This pathway is the key regulator of various cellular processes such as cell differentiation, cell fate, proliferation and stem cell pluripotency. It consists of canonical and non‐canonical pathways, which regulate the gene transcription and cytoskeleton of the cell. Aberrant expression of Wnt signalling pathway was determined in various types of cancers, including a lethal subtype of breast cancer known as triple‐negative breast cancer (TNBC) [46]. Moreover, the dysregulation of canonical pathways was observed in breast cancer [47].

AL022324.3 and Z99916.3 were reported as clone‐based genes acting as lncRNAs in the Ensemble genome database [63]. Their molecular and functional annotations are not reported yet.

This analysis showed that aberrantly expressed lncRNAs and their target mRNAs played important regulatory roles in the biological pathways which lead to cancer upon deregulation. They were significantly differentially expressed and co‐expressed in breast cancer pathways and were also identified as the key regulators in breast cancer progression and metastasis. These integrated lncRNAs‐mRNAs on further investigation and analysis can be used as the potential biomarkers and therapeutic targets for breast cancer in future.

For evaluation of the significant DEGs and confirmation of their roles in BC, two mRNA and two lncRNA datasets from the microarray platform were analysed. The mRNA data of BC provided TAF5L, CYP4F3 and also involves AL022324.4, a contig sequence from one of the target genes AL022324.3 among significantly differentially expressed genes which were also reported in this research as biomarker target mRNAs. The lncRNA datasets along with other DEGs provided the lncRNAs located nearest to those 3 biomarker long non‐coding RNAs retrieved in this study. The exact locations of the biomarker lncRNAs were not observed in these datasets, which might be due to the fact that lncRNA dataset GSE80266 analysed in our study comprised novel lncRNAs with generalised BC samples without any specific conditions. Moreover, it was re‐annotated on the basis of highest sequence score. Furthermore, validation of the gene annotation retrieved from the results of analysed datasets was also performed through TCGA. The TCGA results support findings of the current study. Furthermore, annotations for one of the datasets used for validation GSE113851 were provided already and the other dataset GSE119233 was from Basal‐like Breast Cancer, while this study was focussed on the generalized BC dataset.

The overall analysis supports the results of our research that target mRNAs can serve as potential biomarkers along with the lncRNAs, which are discovered as novel biomarkers and can serve as therapeutic targets in breast cancer.

## 5 CONCLUSION

This study demonstrates that significantly differentially expressed and co‐expressed genes can be used as potential biomarkers and therapeutic targets as they are important regulators of cancer pathways. Advancements in complete molecular and functional annotation of lncRNAs are required. Further progress in lncRNA‐mRNA interactions could be explored through molecular analysis and qRT‐PCR. This therapeutic discovery can be enhanced by identifying candidate biomarkers that can be targeted in cancer therapy.

## Supporting information

Supplementary Material

Supplementary Material

Supplementary Material

Supplementary Material

Supplementary Material

Supplementary Material

Supplementary Material

Supplementary Material

Supplementary Material

Supplementary Material

Supplementary Material

Supplementary Material

Supplementary Material

## References

[1] Ferlay, J. , et al.: Cancer incidence and mortality worldwide: sources, methods and major patterns in globocan 2012. Int. J. Canc. 136(5) (2015) 10.1002/ijc.29210 25220842

[2] Dai, X. , et al.: Cancer hallmarks, biomarkers and breast cancer molecular subtypes. J. Cancer. 7(10), 1281–1294 (2016) 27390604 10.7150/jca.13141 PMC4934037

[3] Yersal, O. , Barutca, S. : Biological subtypes of breast cancer: Prognostic and therapeutic implications. Wjco. 5(3), 412 (2014) 25114856 10.5306/wjco.v5.i3.412 PMC4127612

[4] Lippman, M. , Bolan, G. , Huff, K. : The effects of glucocorticoids and progesterone on hormone‐responsive human breast cancer in long‐term tissue culture. Canc. Res. 36(12), 4602–4609 (1976) 1000505

[5] Shen, X. , et al.: Identification of novel long non‐coding rnas in triple‐negative breast cancer. Oncotarget. 6(25), 21730–21739 (2015) 26078338 10.18632/oncotarget.4419 PMC4673299

[6] Su, X. , et al.: Comprehensive analysis of long non‐coding rnas in human breast cancer clinical subtypes. Oncotarget. 5(20), 9864–9876 (2014) 25296969 10.18632/oncotarget.2454 PMC4259443

[7] Zhao, W. , Luo, J. , Jiao, S. : Comprehensive characterisation of cancer subtype associated long non‐coding rnas and their clinical implications. Sci Rep. 4, 6591 (2014) 25307233 10.1038/srep06591 PMC4194441

[8] Huarte, M. , et al.: A large intergenic noncoding rna induced by p53 mediates global gene repression in the p53 response. Cell. 142(3), 409–419 (2010) 20673990 10.1016/j.cell.2010.06.040 PMC2956184

[9] Gutschner, T. , Diederichs, S. : The hallmarks of cancer. RNA Biol. 9(6), 703–719 (2012) 22664915 10.4161/rna.20481 PMC3495743

[10] Vance, K.W. , Ponting, C.P. : Transcriptional regulatory functions of nuclear long noncoding rnas. Trends Genet. 30(8), 348–355 (2014) 24974018 10.1016/j.tig.2014.06.001 PMC4115187

[11] Krystal, G.W. , Armstrong, B.C. , Battey, J.F. : N‐myc mrna forms an rna‐rna duplex with endogenous antisense transcripts. Mol. Cell Biol. 10(8), 4180–4191 (1990) 1695323 10.1128/mcb.10.8.4180-4191.1990 PMC360949

[12] Faghihi, M.A. , Wahlestedt, C. : Regulatory roles of natural antisense transcripts. Nat. Rev. Mol. Cell Biol. 10(9), 637–643 (2009) 19638999 10.1038/nrm2738 PMC2850559

[13] Carrieri, C. , et al.: Long non‐coding antisense rna controls uchl1 translation through an embedded sineb2 repeat. Nature. 491(7424), 454–457 (2012) 23064229 10.1038/nature11508

[14] Gong, C. , Maquat, L.E. : lncRNAs transactivate STAU1‐mediated mRNA decay by duplexing with 3′ UTRs via Alu elements. Nature. 470(7333), 284–288 (2011) 21307942 10.1038/nature09701 PMC3073508

[15] Van.Grembergen, O. , et al.: Portraying breast cancers with long noncoding rnas. Sci. Adv. 2(9) e1600220 (2016) 27617288 10.1126/sciadv.1600220 PMC5010371

[16] Cheetham, S.W. , et al.: Long noncoding rnas and the genetics of cancer. Br. J. Cancer. 108(12), 2419–2425 (2013) 23660942 10.1038/bjc.2013.233 PMC3694235

[17] Clark, M.B. , et al.: Genome‐wide analysis of long noncoding rna stability. Genome Res. 22(5), 885–898 (2012) 22406755 10.1101/gr.131037.111 PMC3337434

[18] Gibb, E.A. , Brown, C.J. , Lam, W.L. : The functional role of long non‐coding rna in human carcinomas. Mol. Canc. 10(1), 38 (2011) 10.1186/1476-4598-10-38 PMC3098824 21489289

[19] Anders, S. , Huber, W. : Differential expression analysis for sequence count data. Genome Biol. 11(10), R106 (2010) 20979621 10.1186/gb-2010-11-10-r106 PMC3218662

[20] Langfelder, P. , Horvath, S. : Wgcna: an r package for weighted correlation network analysis. BMC Bioinf. 9(1), 559 (2008) 10.1186/1471-2105-9-559 PMC2631488 19114008

[21] Trapnell, C. , Pachter, L. , Salzberg, S.L. : Tophat: discovering splice junctions with rna‐seq. Bioinformatics. 25(9), 1105–1111 (2009) 19289445 10.1093/bioinformatics/btp120 PMC2672628

[22] Quinlan, A.R. , Hall, I.M. : Bedtools: a flexible suite of utilities for comparing genomic features. Bioinformatics. 26(6), 841–842 (2010) 20110278 10.1093/bioinformatics/btq033 PMC2832824

[23] Wang, L. , et al.: Cpat: coding‐potential assessment tool using an alignment‐free logistic regression model, Nucleic acids research. 41(6), pp. e74–e74 (2013) 23335781 10.1093/nar/gkt006 PMC3616698

[24] Benjamini, Y. , Hochberg, Y. : Controlling the false discovery rate: a practical and powerful approach to multiple testing. J. Roy. Stat. Soc. B. 57, 289–300 (1995)

[25] Changyong, F. , et al.: Log‐transformation and its implications for data analysis. Shanghai Arch. Psychiatry. 26(2), 105 (2014) 25092958 10.3969/j.issn.1002-0829.2014.02.009 PMC4120293

[26] Ritchie, M.E. , et al.: Limma powers differential expression analyses for rna‐sequencing and microarray studies, Nucleic Acids Res. 43, (7), pp. e47 (2015) 25605792 10.1093/nar/gkv007 PMC4402510

[27] Kent, W.J. : BLAT‐‐‐The BLAST‐like alignment tool. Genome Res. 12(4), 656–664 (2002) 11932250 10.1101/gr.229202 PMC187518

[28] Djebali, S. , et al.: Landscape of transcription in human cells. Nature. 489(7414), 101 (2012) 22955620 10.1038/nature11233 PMC3684276

[29] Sultan, M. , et al.: A global view of gene activity and alternative splicing by deep sequencing of the human transcriptome. Science. 321(5891), 956–960 (2008) 18599741 10.1126/science.1160342

[30] Ferreira, P.G. , et al.: Transcriptome characterisation by rna sequencing identifies a major molecular and clinical subdivision in chronic lymphocytic leukemia. Genome Res. 24(2), 212–226 (2014) 24265505 10.1101/gr.152132.112 PMC3912412

[31] A Fas tqc, S. : A Quality Control Tool For High Throughput Sequence Data (2010). Available onlineat: http://www.bioinformatics.babraham.ac.uk/projects/fastqc

[32] Bolger, A.M. , Lohse, M. , Usadel, B. : Trimmomatic: a flexible trimmer for illumina sequence data. Bioinformatics. 30(15), 2114–2120 (2014) 24695404 10.1093/bioinformatics/btu170 PMC4103590

[33] Langmead, B. , et al.: Ultrafast and memory‐efficient alignment of short dna sequences to the human genome. Genome Biol. 10(3), R25 (2009) 19261174 10.1186/gb-2009-10-3-r25 PMC2690996

[34] Giardine, B. , et al.: Galaxy: a platform for interactive large‐scale genome analysis. Genome Res. 15(10), 1451–1455 (2005) 16169926 10.1101/gr.4086505 PMC1240089

[35] Robinson, M.D. , McCarthy, D.J. , Smyth, G.K. : edger: a bioconductor package for differential expression analysis of digital gene expression data. Bioinformatics. 26(1), 139–140 (2010) 19910308 10.1093/bioinformatics/btp616 PMC2796818

[36] Kornienko, A.E. , et al.: Gene regulation by the act of long non‐coding rna transcription. BMC Biol. 11(1), 59 (2013) 23721193 10.1186/1741-7007-11-59 PMC3668284

[37] Kanehisa, M. , Goto, S. : Kegg: kyoto encyclopaedia of genes and genomes. Nucleic Acids Res. 28(1), 27–30 (2000) 10592173 10.1093/nar/28.1.27 PMC102409

[38] Belinky, F. , et al.: Pathcards: Multi‐Source Consolidation of Human Biological Pathways, Database, vol. 2015, p. bav006 (2015). 10.1093/database/bav006 PMC4343183 25725062

[39] Colaprico, A. , et al.: Tcgabiolinks: an r/bioconductor package for integrative analysis of tcga data. Nucleic Acids Res. 44(8), e71 (2016) 26704973 10.1093/nar/gkv1507 PMC4856967

[40] Soneson, C. , Delorenzi, M. : A comparison of methods for differential expression analysis of rna‐seq data. BMC Bioinf. 14(1), 91 (2013) 10.1186/1471-2105-14-91 PMC3608160 23497356

[41] Jan, R. , et al.: Loss of pigment epithelium‐derived factor: a novel mechanism for the development of endocrine resistance in breast cancer. Breast Cancer Res. 14(6), R146 (2012) 23151593 10.1186/bcr3356 PMC3906603

[42] Guan, M. , et al.: Inhibition of glioma invasion by overexpression of pigment epithelium‐derived factor. Cancer Gene Ther. 11(5), 325–332 (2004) 15044958 10.1038/sj.cgt.7700675

[43] Trzaskowski, B. , et al.: Action of molecular switches in GPCRs ‐ theoretical and experimental studies. Cmc. 19(8), 1090–1109 (2012) 10.2174/092986712799320556 PMC3343417 22300046

[44] Filardo, E.J. : Epidermal growth factor receptor (egfr) transactivation by oestrogen via the g‐protein‐coupled receptor, gpr30: a novel signalling pathway with potential significance for breast cancer. J. Steroid Biochem. Mol. Biol. 80(2), 231–238 (2002) 11897506 10.1016/s0960-0760(01)00190-x

[45] Thomas, P. , et al.: Identity of an oestrogen membrane receptor coupled to a g protein in human breast cancer cells. Endocrinology. 146(2), 624–632 (2005) 15539556 10.1210/en.2004-1064

[46] Pohl, S.‐G. , et al.: Wnt signalling in triple‐negative breast cancer. Oncogenesis. 6(4), e310–e310 (2017) 28368389 10.1038/oncsis.2017.14 PMC5520491

[47] Boras Granic, K. , Wysolmerski, J.J. : Wnt signalling in breast organogenesis. Organogenesis. 4(2), 116–122 (2008) 19279723 10.4161/org.4.2.5858 PMC2634257

[48] Edson, K. , Rettie, A. : CYP4 enzymes as potential drug targets: focus on enzyme multiplicity, inducers and inhibitors, and therapeutic modulation of 20‐ hydroxyeicosatetraenoic acid (20‐HETE) synthase and fatty acid ω‐ hydroxylase activities. Ctmc. 13(12), 1429–1440 (2013) 10.2174/15680266113139990110 PMC4245146 23688133

[49] Hanahan, D. , Weinberg, R.A. : Hallmarks of cancer: the next generation. Cell. 144(5), 646–674 (2011) 21376230 10.1016/j.cell.2011.02.013

[50] Hensley, C.T. , Wasti, A.T. , DeBerardinis, R.J. : Glutamine and cancer: cell biology, physiology, and clinical opportunities. J. Clin. Invest. 123(9), 3678–3684 (2013) 23999442 10.1172/JCI69600 PMC3754270

[51] Cheng, T. , et al.: Pyruvate carboxylase is required for glutamine‐independent growth of tumour cells. Proc. Natl. Acad. Sci. Unit. States Am. 108(21), 8674–8679 (2011) 10.1073/pnas.1016627108 PMC3102381 21555572

[52] Ling, H. , et al.: Junk dna and the long non‐coding rna twist in cancer genetics. Oncogene. 34(39), 5003–5011 (2015) 25619839 10.1038/onc.2014.456 PMC4552604

[53] Gupta, R.A. , et al.: Long non‐coding RNA HOTAIR reprograms chromatin state to promote cancer metastasis. Nature. 464(7291), 1071–1076 (2010) 20393566 10.1038/nature08975 PMC3049919

[54] Volinia, S. , Croce, C.M. : Prognostic microrna/mrna signature from the integrated analysis of patients with invasive breast cancer. Proc. Natl. Acad. Sci. Unit States Am. 110(18), 7413–7417 (2013) 10.1073/pnas.1304977110 PMC3645522 23589849

[55] O’Leary, V.B. , et al.: Particle, a triplex‐forming long ncrna, regulates locus‐specific methylation in response to low‐dose irradiation. Cell Rep. 11(3), 474–485 (2015) 25900080 10.1016/j.celrep.2015.03.043

[56] Schiltz, R.L. , Nakatani, Y. : The pcaf acetylase complex as a potential tumour suppressor. Biochim. Biophys. Acta Rev. Canc. 1470(2), M37–M53 (2000) 10.1016/s0304-419x(99)00037-2 10722926

[57] Okumura, K. , et al.: Pcaf modulates pten activity. J. Biol. Chem. 281(36), 26562–26568 (2006) 16829519 10.1074/jbc.M605391200

[58] Shubbar, E. , et al.: High levels of *γ*‐glutamyl hydrolase (ggh) are associated with poor prognosis and unfavourable clinical outcomes in invasive breast cancer. BMC Canc. 13(1), 47 (2013) 10.1186/1471-2407-13-47 PMC3576262 23374458

[59] Vainio, P. , et al.: Arachidonic acid pathway members pla2g7, hpgd, ephx2, and cyp4f8 identified as putative novel therapeutic targets in prostate cancer. Am. J. Pathol. 178(2), 525–536 (2011) 21281786 10.1016/j.ajpath.2010.10.002 PMC3128506

[60] Stewart, P.A. , et al.: Differentially expressed transcripts and dysregulated signalling pathways and networks in sAfrican American breast cancer. PloS One. 8(12) e82460 (2013) 24324792 10.1371/journal.pone.0082460 PMC3853650

[61] Hammamieh, R. , et al.: Control of the growth of human breast cancer cells in culture by manipulation of arachidonate metabolism. BMC Canc. 7(1), 138 (2007) 10.1186/1471-2407-7-138 PMC1940258 17651499

[62] Vinayagam, A. , et al.: A directed protein interaction network for investigating intracellular signal transduction. Sci. Signal. 4(189), rs8–rs8 (2011) 21900206 10.1126/scisignal.2001699

[63] Kersey, P.J. , et al.: Ensembl genomes: extending ensembl across the taxonomic space. Nucleic Acids Res. 38(suppl_1), D563–D569 (2009) 19884133 10.1093/nar/gkp871 PMC2808935

