# Supplementary material for: Long non‐coding RNAs and their targets as potential biomarkers in breast cancer
Source: IET Syst Biol. 2021 May 15;15(5):137–47. doi: 10.1049/syb2.12020 (PMC8675856; doi:10.1049/syb2.12020)
Supplement: Supplementary file 1 — Supplementary Material [file SYB2-15-137-s004.pdf]

1. Supplementary Figures

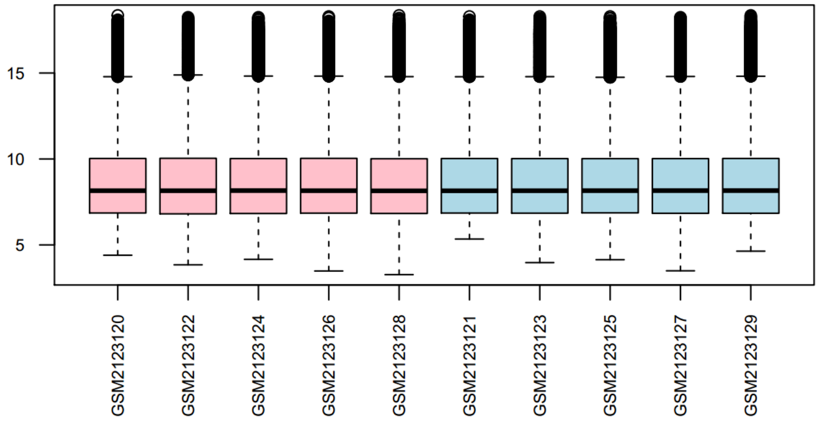

Figure 1: **Boxplot dataset GSE80266.**

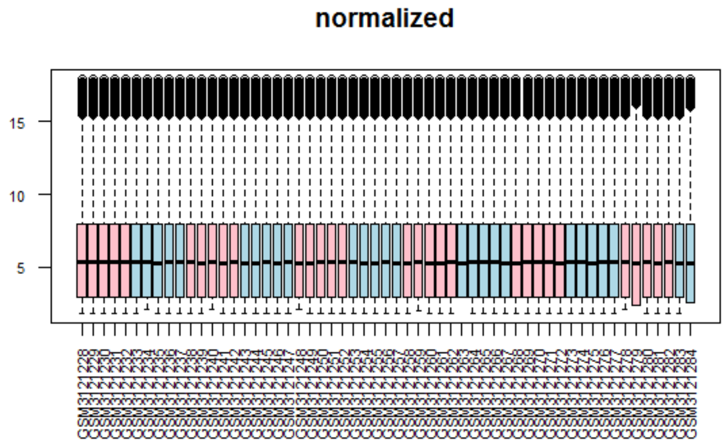

Figure 2: **Boxplot dataset GSE113851.**

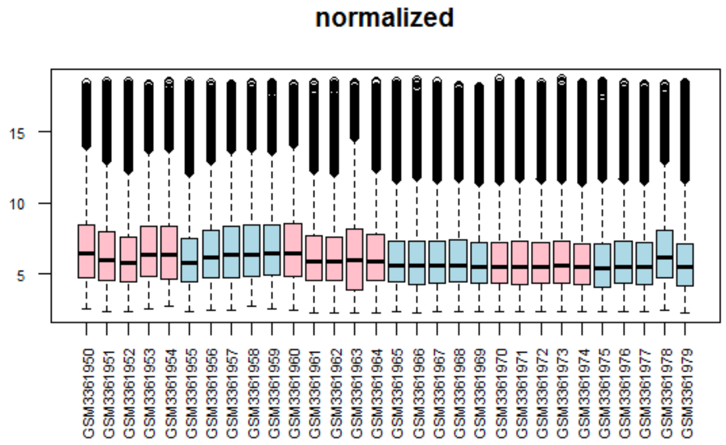

Figure 3: **Boxplot dataset GSE119233.**

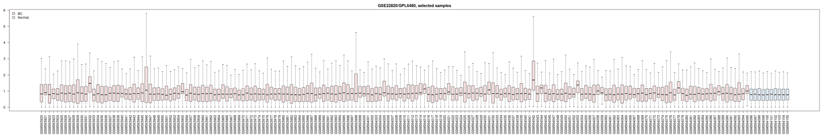

Figure 4: **Boxplot dataset GSE22820.**

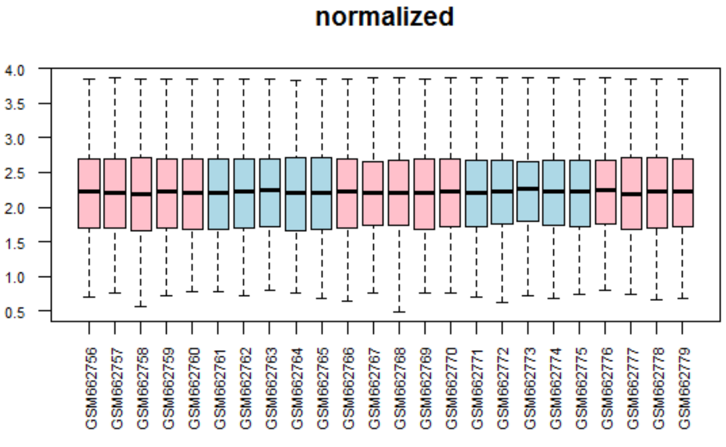

Figure 5: **Boxplot dataset GSE26910.**

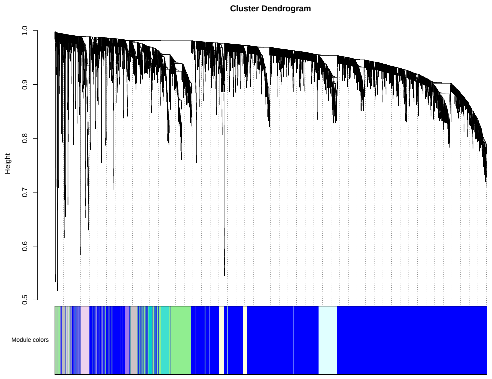

Figure 6: **Cluster Dendogram of dataset GSE113851.**Branches of the dendrogram correspond to modules with correlated genes represented by different colors

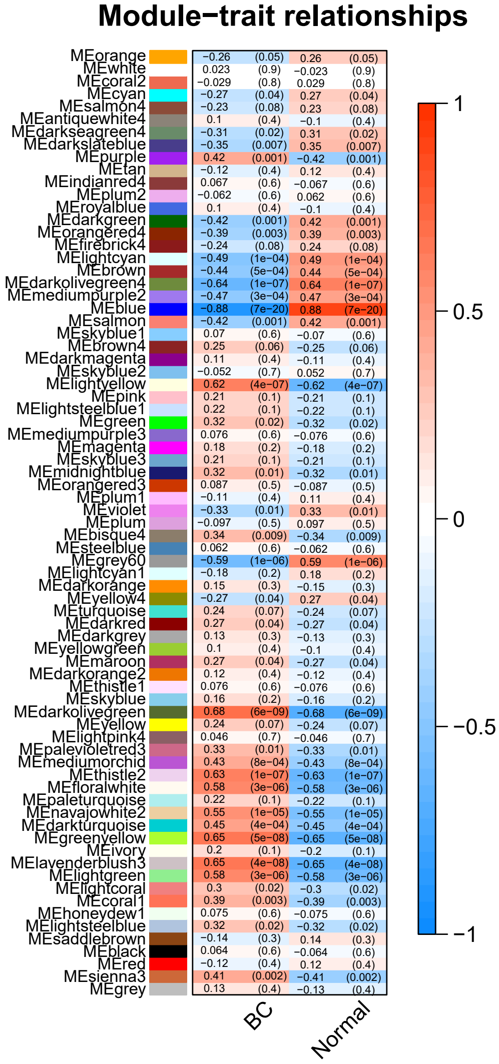

Figure 7: **Module-Trait relationship of GSE113851.** There are 75 modules, along with a color name, correlation and p-values. Module with dark red color in the heat map, i.e. MEblue shows a high correlation of 88% along with its significant p-value of 7e-20

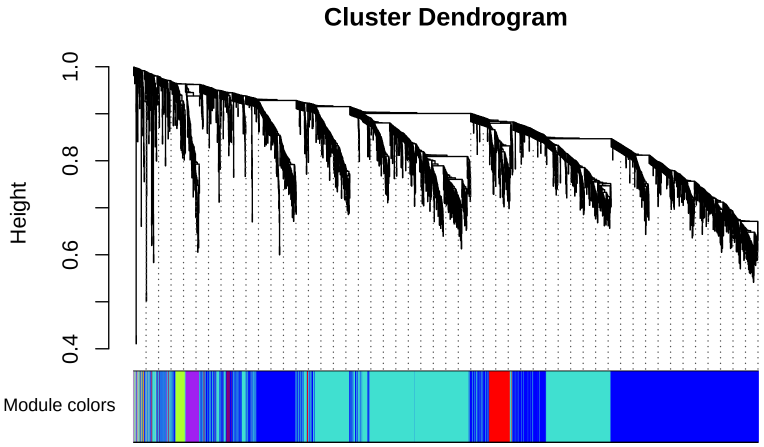

Figure 8: **Cluster Dendogram of dataset GSE119233.**Branches of the dendrogram correspond to modules with correlated genes represented by different colors

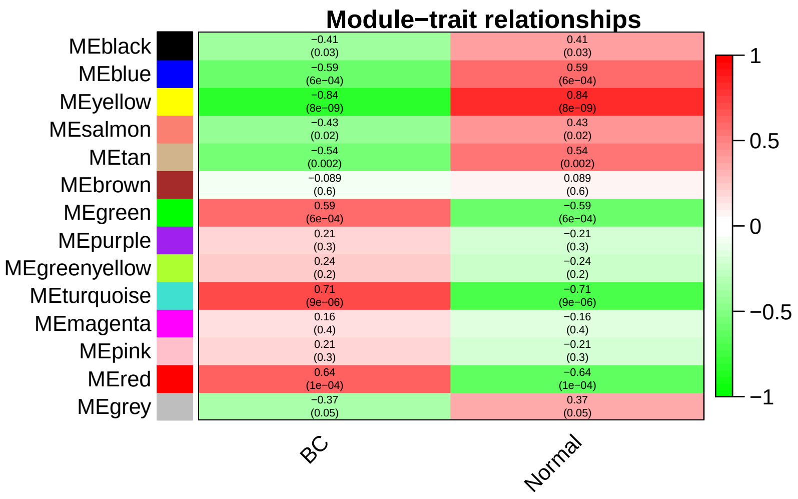

Figure 9: **Module-Trait relationship of GSE119233.** Total 14 modules along with a color name, correlation and respective p-values are represented in R plot. Module with dark red color in the heat map, i.e. MEyellow and shows a high correlation of 84% along with its significant p-value of 8e-09

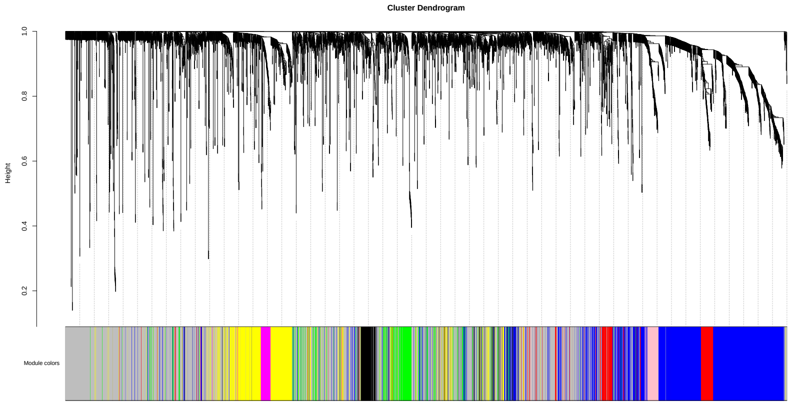

Figure 10: **Cluster Dendrogram of dataset GSE22820.**Branches of the dendrogram correspond to modules with correlated genes represented by different colors

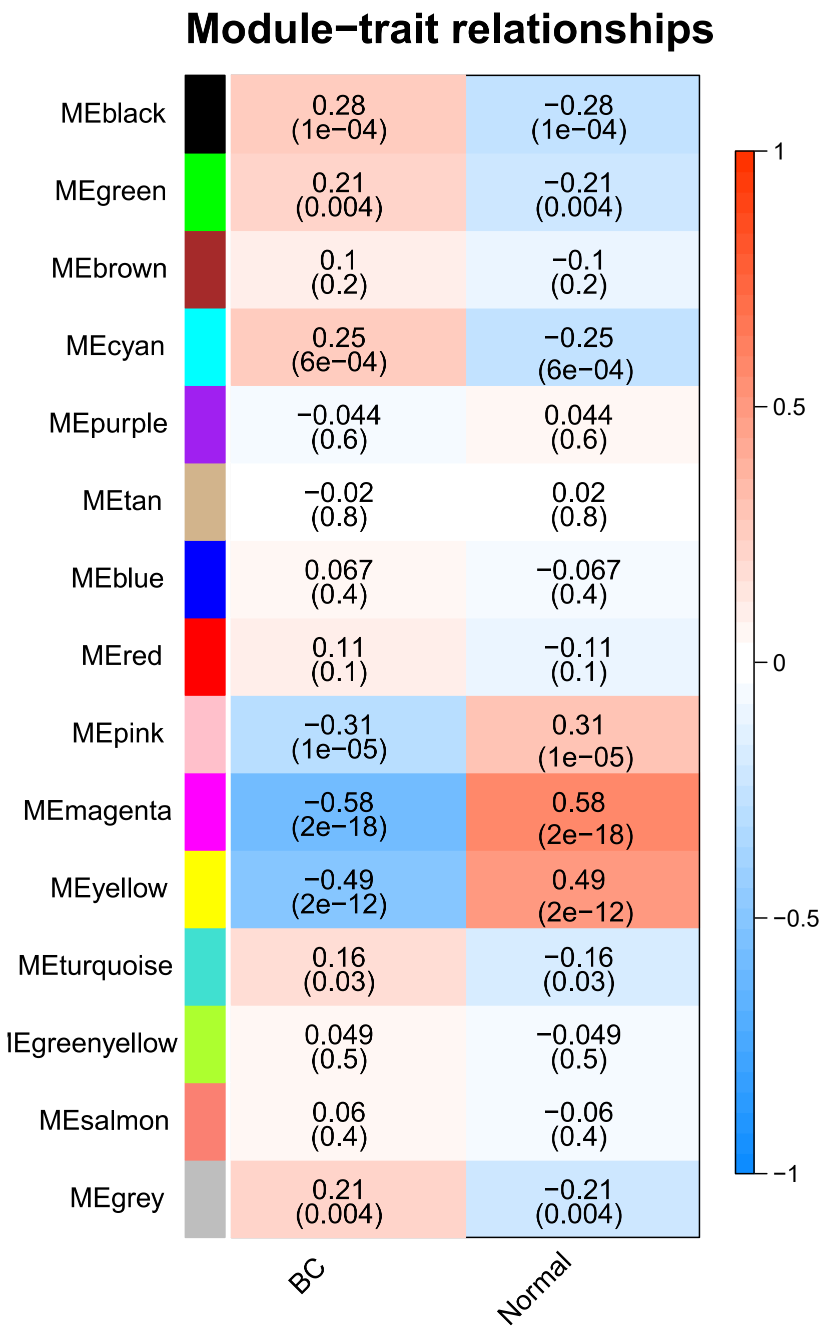

Figure 11: **Module-Trait relationship of GSE22820.** The heat map shows total 15 modules correlated with external traits, i.e. breast cancer and normal. Moreover, MEmagenta was the only module shown in dark red color to hold a 58% correlation with a least p-value of 2e -18

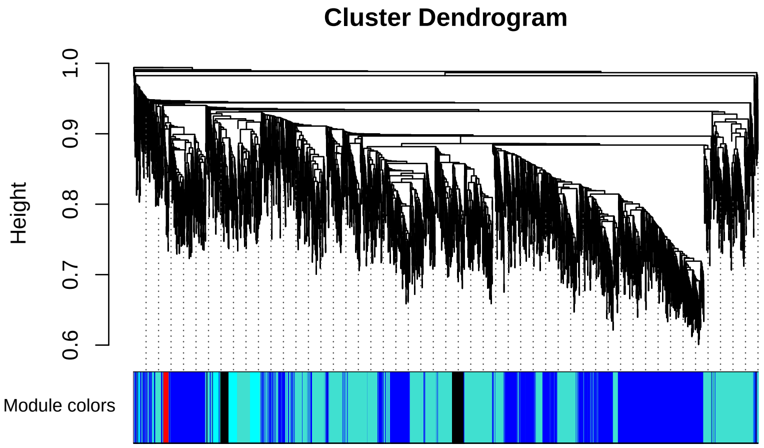

Figure 12: **Cluster Dendrogram of dataset GSE26910.**Branches of the dendrogram correspond to modules with correlated genes represented by different colors

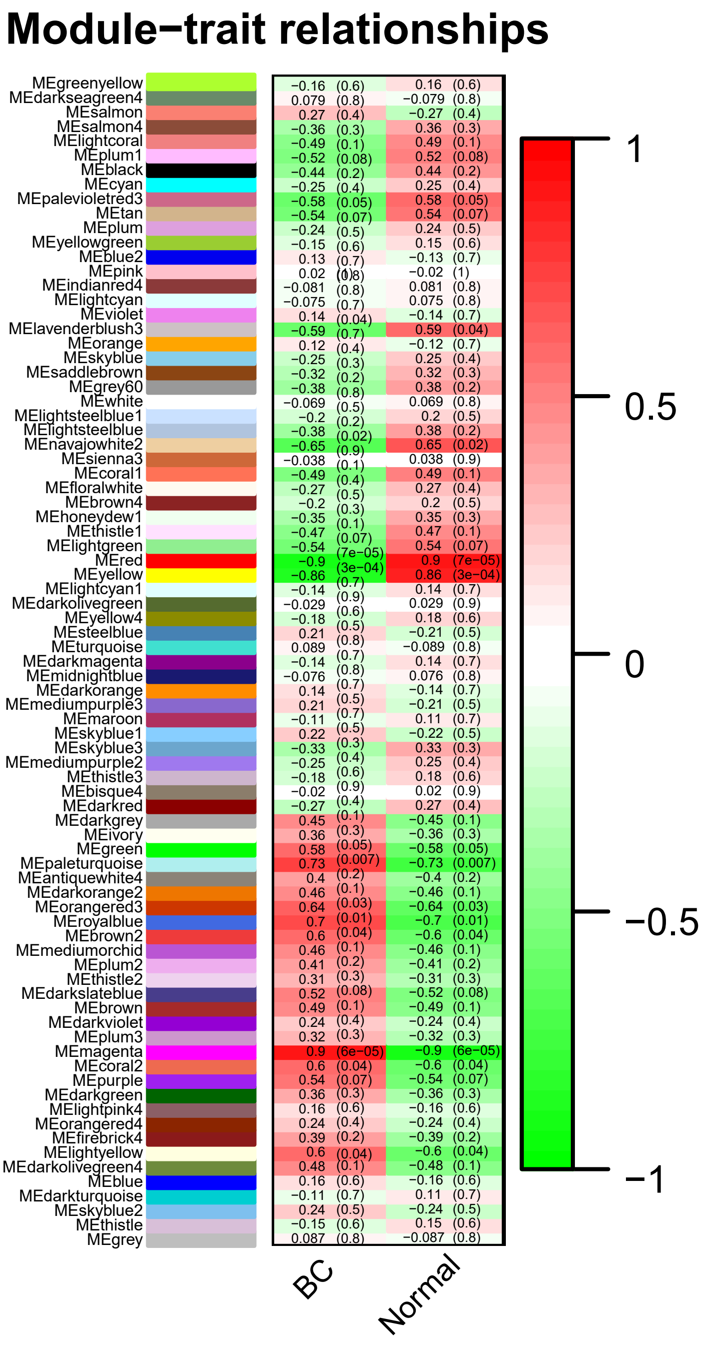

Figure 13: **Module-Trait relationship of GSE26910.** The heat map displays total 81 modules significantly correlated with external traits, i.e. breast cancer and normal. Only two modules MEmagenta and MEred shown in dark red color hold a significant correlation with a least p-value
